# Health Outcome Measures in Atopic Dermatitis: A Systematic Review of Trends in Disease Severity and Quality-of-Life Instruments 1985–2010

**DOI:** 10.1371/journal.pone.0017520

**Published:** 2011-04-13

**Authors:** Balvinder Rehal, April Armstrong

**Affiliations:** Department of Dermatology, University of California Davis, Davis, California, United States of America; Copenhagen University Hospital Gentofte, Denmark

## Abstract

**Background:**

A number of disease-severity and quality-of-life (QoL) instruments have emerged in atopic dermatitis (AD) in the last decade.

**Objectives:**

To identify trends in outcomes instruments used in AD clinical trials and to provide a useful summary of the dimensions and validation studies for the most commonly used measures.

**Method:**

All randomized control trials (RCTs) from 1985 to 2010 in the treatment of AD were examined.

**Results:**

Among the 791 RCTs reviewed, we identified 20 disease-severity and 14 QoL instruments. Of these outcomes instruments, few have been validated. SCORAD, EASI, IGA and SASSAD were the most commonly used disease-severity instruments and CDLQI, DFI, DLQI and IDQOL were the most frequently used QoL measures.

**Limitations:**

The small number of RCTs using QoL scales makes identifying trends for QoL instruments difficult.

**Conclusion:**

Overall, there is an increase in the use of disease-severity and QoL instruments in AD clinical trials.

## Introduction

Atopic dermatitis is a chronic, inflammatory skin disease that affects patients' physical and psychosocial wellbeing. The burden of atopic dermatitis has been documented in the medical literature [1], [2]. Patients suffering from atopic dermatitis often experience embarrassment from the skin lesions, and severe disease can adversely affect social interactions and personal relationships. The symptoms of atopic dermatitis, notably pruritus, can be intractable and lead to significant emotional distress and sleep loss [3].

Despite continuing efforts in developing new treatments for atopic dermatitis, scarce literature exists that evaluates disease-severity and quality-of-life (QoL) outcome measures in AD [4], [5], [6], [7]. This systematic review examines the trends in outcomes instruments, specifically disease-severity and QoL instruments, in randomized controlled trials (RCT) in the treatment of AD published between 1985 to 2010. We discuss the most frequently used disease-severity and QoL measures in terms of their dimensions (aspects of AD that the instrument measures) and validation studies that have supported their increased use in clinical trials.

## Methods

To examine the disease and QoL outcome measures used in atopic dermatitis trials, we conducted a systematic review of RCTs for AD from 1985 to 2010 in the U.S. National Library of Medicine using the Medline search engine and in the electronic database, Scopus, which includes the EMBASE database. In Medline we applied the Medical Subject Headings search terms “atopic dermatitis” and “treatment” and limited the search to human RCTs published in the English language from January 1, 1985 to July 14, 2010. In Scopus, we searched for RCTs in atopic dermatitis using the search “TITLE-ABS-KEY(atopic dermatitis) AND (TITLE-ABS-KEY(randomized control trial*) OR TITLE-ABS-KEY(RCT)).”

## Results

**Figure 1:**
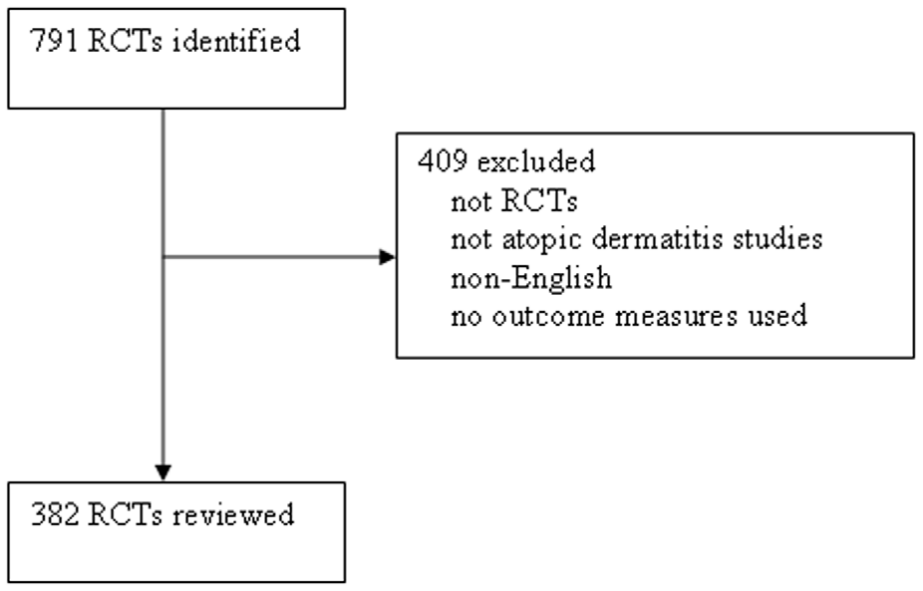
Excluded studies.

In Medline, our search identified an initial group of 552 studies published between 1985 and 20010 in AD. Of these 552 studies, 195 were excluded either because they were not RCTs, not pertaining to atopic dermatitis studies, not in English, or no outcome measures were used (Figure 1). In Scopus our search generated 239 studies from 1985–2010. After cross-referencing the list of studies with our Medline search, we were left with 141 articles. Of these, 116 were excluded for the reasons listed above. After exclusion, 382 studies were reviewed from both Medline and Scopus.

A total of 20 disease-severity scales and 14 QoL scales were used in the RCTs for the treatment of atopic dermatitis. We list the disease-severity instruments and QoL measures in Tables 1 and 2, respectively.

**Table 1:** Severity of disease scales.

Scale	Clinical signs	Disease extent	Subjective Sx	# of studies used in
	Erythema	Edema/papulation	oozing/crusts	excoriation	lichenification	dryness/scaling			
Severity scoring of atopic dermatitis (SCORAD)	*	*	*	*	*		*	*	76
Eczema Area and Severity Index (EASI)	*	*		*	*		*		51
Investigators' Global Assessment (IGA)	*	*	*						41
Six Area, Six Sign Atopic Dermatitis (SASSAD)	*		*	*	*	*	*	*	14
Investigators' Global Atopic Dermatitis Assessment (IGADA)	*	*	*	*	*	*	*		4
Costa et al	*	*	*	*	*	*	*	*	3
Leiciester Sign Score (LSS)	*		*	*	*	*	*		2
Visual Analogue Scale (VAS) pruritus								*	2
Total Severity Score (TSS)	*	*	*	*	*		*		2
Physicians Global Assessment (PGA)	*					*	*		2
Intensity Item Score Aggregate (IISA)	*	*	*	*	*	*	*	*	1
Atopic Dermatitis Severity Index (ADSI)	*		*	*	*		*	*	1
Investigators' Static Global Assessment (ISGA)	*	*	*		*	*		*	1
Nottingham Eczema Severity Score (NESS)							*	*	1
Investigators' Global Assessment Score (IGAS)	*	*						*	1
Dry skin are and severity index (DASI)	*					*	*	*	1
Atopic Dermatitis Area and Severity Index (ADASI)	*	*	*	*	*	*	*		1
Total body severity assessment (TBSA)	*	*	*	*		*	*		1

**Table 2:** Quality of life scales.

Scale	Questions	# Studies used in
	Severity	itching	mood	sleep	dressing/clothes	leisure activities	treatment	Parent mood	Parent sleep	family disruption/tension	
Children's Dermatology Life Quality Index (CDLQI)		*	*	*	*	*	*				13
Dermatology Life Quality Index (DLQI)		*	*		*	*	*				8
Infant's Dermatology Quality of Life (IDQOL)	*	*	*	*	*	*	*				7
Dermatitis Family Impact (DFI)				*		*		*	*	*	6
Parent's Index of Quality of Life in Atopic Dermatitis (PIQoL-AD)					*		*	*	*	*	2
Quality of Life Index for Atopic Dermatitis (QoLIAD)			*		*	*					2
Short Form Health Survey (SF-36)			*		*	*					2
Parents of Children with Atopic Dermatitis (PQoL–AD)								*	*		1
German Instrument for the assessment of Quality of Life in Skin Diseases (DIELH)		*	*			*	*				1
Eczema Disability Index (EDI)						*	*				1
Skindex-29		*	*	*		*					1

The most frequently used disease-severity instruments from 1985 to 2010 were the Severity scoring of atopic dermatitis (SCORAD), Eczema Area and Severity Index (EASI), Investigators' Global Assessment (IGA) and Six Area, Six Sign Atopic Dermatitis (SASSAD) (Table 1). SCORAD was the most frequently used scale; it was used in 113 out of 382 RCTs (30%). The next most frequently used scale was the EASI, which was used in 63 out of 382 RCTs (16%), followed by the IGA that was used in 48 out of 382 RCTs (13%). SASSAD was used in 18 out of 382 RCTs (5%). The four most commonly used scales, SCORAD, EASI, IGA and SASSAD, were used in the majority of RCTs: 242 out of 382 (63%). The remaining 14 scales were used in 57 out of 382 RCTs (15%).

**Figure 2:**
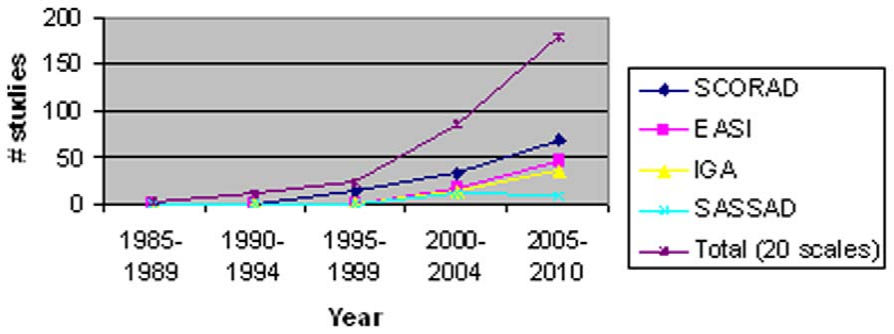
Trends in Disease Severity Instruments 1985–2010.

The trend for disease-severity scales showed that the number of disease-severity instruments used in clinical trials increased dramatically from 1985 to 2010 (Figure 2). Specifically, SCORAD was used in 4% of RCTs from 1985–1997 and 40% RCTs from 1998–2010. SCORAD had its peak usage from 2005 to 2010.

EASI, IGA and SASSAD were also used more commonly from 1985–2010 (Figure 2). From 1985–1997, no RCTs used EASI, IGA or SASSAD. EASI also had its peak usage from 2005 to 2010. To our knowledge, IGA has not been validated to date, but its usage has been nearly identical to that of EASI (Figure 2). Out of the 48 RCTs that used IGA, 32 trials used IGA in conjunction with EASI (67%).

**Figure 3:**
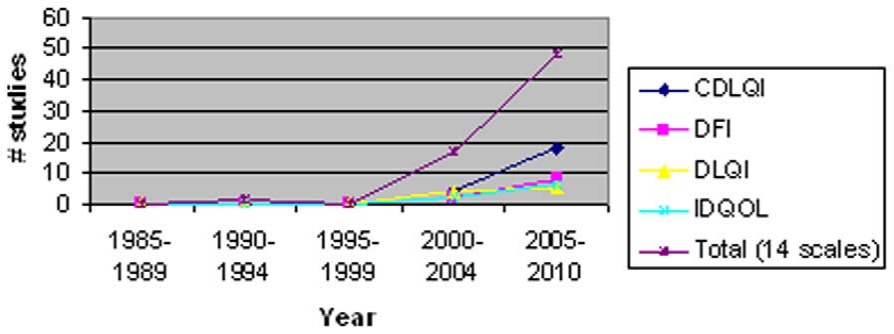
Trends in Quality of Life Instruments 1985–2010.

Among the 382 RCTs, 67 studies employed QoL instruments. Of the studies that used QoL outcomes measures, the Children's Dermatology Life Quality Index (CDLQI) was the most frequently used (33%), followed by the Dermatitis Family Impact (DFI) (15%), the Dermatology Life Quality Index (DLQI) (13%) and the Infant's Dermatology Life Quality Index.

(IDQOL) (12%) (Table 2). Overall, the use of QoL scales in RCTs has increased from 1985 to 2010 (Figure 3). None of the four most commonly used QoL instruments, CDLQI, DFI, DLQI or IDQOL were used between 1985–1997.

### Common Disease-Severity Scales in Atopic Dermatitis: Dimensions and Evidence for Validation

#### Severity Scoring of Atopic Dermatitis (SCORAD)

From 1985 to 2010, SCORAD was the most widely used disease-severity scale in atopic dermatitis. SCORAD was used in 113 out of the 382 studies that met our search criteria (30%). It was developed in 1993 by the European Task Force on Atopic Dermatitis [8]. The SCORAD index uses the rule of nines to assess disease extent and evaluates five clinical characteristics to determine disease severity: (1) erythema, (2) edema/papulation, (3) oozing/crusts, (4) excoriation and (5) lichenification. SCORAD also assesses subjective symptoms of pruritus and sleep loss with Visual Analogue Scales (VAS) [8]. These three aspects: extent of disease, disease severity and subjective symptoms combine to give a maximum possible score of 103. Although it is a combined score, the three aspects can be separated and used individually if necessary. Of all the severity scales used in atopic dermatitis, it is the most widely validated disease-severity instrument [9]. SCORAD has been found to be valid and reliable, and it has shown excellent agreement with global assessments of disease severity [8], [9], [10], [11], [12], [13], [14], [15], [16], [17], [18], [19], [20], [21], [22]. However, some studies have shown interobserver variation in scoring lichenification and extent of disease [8], [9], [10], [18], [20], [23].

#### Eczema Area and Severity Index (EASI)

EASI was the second most commonly used scale in our review of the literature. It was used in 63 out of 382 RCTs (16%). EASI was developed by modifying the PASI (Psoriasis Area and Severity Index), a widely accepted and standardized scoring system for psoriasis [24]. EASI assesses extent of disease at four body sites and measures four clinical signs: (1) erythema, (2) induration/papulation, (3) excoriation, and (4) lichenification each on a scale of 0 to 3. EASI confers a max score of 72 [25]. EASI evaluates two dimensions of atopic dermatitis: disease extent and clinical signs. Unlike the SCORAD, it does not assess symptoms like pruritus and sleep loss. Some investigators express that subjective symptoms may be the most important marker for assessing patient morbidity and they may also be a good indicator for disease severity. In a large validation study with a cohort of 1550 pediatric patients, EASI was found to have excellent validity, internal consistency and sensitivity to change [26]. While EASI is a valid and reliable instrument, most interobserver variability lies in the dimension of induration/papulation [13], [21], [25], [26], [27].

#### Investigators' Global Assessment (IGA)

IGA was the third most common scale, used in 48 out of 382 RCTs (13%). IGA allows investigators to assess overall disease severity at one given time point, and it consists of a 6-point severity scale from clear to very severe disease (0 = clear, 1 = almost clear, 2 = mild disease, 3 = moderate disease, 4 = severe disease and 5 = very severe disease) [28]. IGA uses clinical characteristics of erythema, infiltration, papulation, oozing and crusting as guidelines for the overall severity assessment [28]. To our knowledge, IGA has not been validated as an outcome measure [7]. However, IGA has been used to validate other outcome scales as one “gold standard.” [9], [26] While the combined use of IGA with another validated scale does not make IGA itself a stand-alone, validated instrument, IGA appears to correlate well with the EASI and is considered an instrument with reasonable face validity. Potential weaknesses of IGA include lack of responsiveness and discrimination for disease severity and lack of subjective symptoms.

#### Six-Area, Six-Sign Atopic Dermatitis (SASSAD)

SASSAD was used in 18 out of 382 RCTs (5%), which ranks SASSAD as the fourth most commonly used scale. SASSAD assesses six clinical signs of disease severity (erythema, exudation, dryness, cracking, excoriation, and lichenification) at six body sites (head/neck, trunk, arms, hands, legs and feet). Each clinical sign on a given body site is graded on a scale of 0–3, and the scale confers a maximum score of 108 [29]. SASSAD does not assess subjective symptoms. The SASSAD is sensitive to changes in topical steroid requirements, pruritus, and sleep loss [30], [31], [32], [33], [34]. In a small reliability study involving 6 patients, there was interobserver variation for dryness and lichenification [35].

### Quality-of-Life Instruments in Atopic Dermatitis: Dimensions and Evidence for Validation

#### Children's Dermatology Life Quality Index (CDLQI)

CDLQI was the most common QoL instrument from our search, used in 22 out of 382 RCTs (6%). Drs. Lewis-Jones and Finlay developed and validated CDLQI in 1995, with the purpose of measuring the QoL in children with skin disease [36]. The questionnaire was designed for children ages 4 through 16. CDLQI is completed by the child with the help of an adult if necessary, preferably a parent. The questionnaire consists of 10 questions that encompass different aspects of a child's life that could be affected by their skin disease. The instrument includes physical symptoms, such as itching and sleep loss, as well as psychosocial questions regarding friendships, bullying, school performance, sports participation, and enjoyment of vacation. The questions are graded from 0–3, with a possible maximum score of 30 with higher scores representing worse QoL. [36]. In the initial validation study, children with atopic eczema accounted for 20% of all patients [36]. To determine test-retest repeatability, CDLQI was used in a population of children without skin disease [36].

Since its validation, CDLQI has been used in numerous studies to determine the effectiveness of interventions in children with AD [37], [38]. CDLQI has been translated and validated in Cantonese [39], [40], [41]. A cartoon version of CDLQI was validated in 2003, which appears to be quicker and preferred by children [42].

#### Dermatitis Family Impact (DFI)

The DFI questionnaire was used in 10 out of 382 RCTs (2.6%), making it the second most common quality of life scale used in our review. It was developed in 1998 by Drs. Lawson, Lewis-Jones, Finlay, Reid and Owens to help measure how family life is affected by a child suffering from atopic dermatitis [43]. It is designed to be completed by a caretaker of the child, usually a parent, and consists of 10 questions related to housework, food preparation and feeding, sleep, family leisure activity, shopping, expenditure, fatigue, emotional distress and relationships [43]. Each question is graded from 0–3 with a maximum possible score of 30. DFI has been found to be valid, reliable, and sensitive to change in multiple studies [6], [39], [43], [44], [45], [46], [47], [48]. Two studies that assessed validity of the instrument were based on using separate components of the DFI, as opposed to using the total score as was originally intended by creators of the scale [43], [46]. DFI has also been validated in Malay and Portuguese [39], [44], [45], [48].

#### Dermatology Life Quality Index (DLQI)

DLQI was the third most common QoL scale that was used in 9 out of 382 RCTs (2.4%) in our review. DLQI was developed in 1994 by Drs. Finlay and Khan to measure quality of life in routine clinical practice in adults over age of 18 [49]. DLQI is a 10-item questionnaire that inquires about skin symptoms, feelings of embarrassment, and how skin disease has affected day-to-day activities, working and social life. Similar to CDLQI, each question on DLQI is scored from 0 to 3 with a maximum score of 30 and high scores representing worse QoL.

Both DLQI and CDLQI are specialty-specific but not disease-specific QoL instruments. In the original article by Finlay and Khan, patients with atopic dermatitis had the worst QoL as measured by DLQI compared to the other skin diseases assessed in the study [49]. DLQI has been extensively validated in multiple studies [49], [50], [51]. A 10-year review of the literature found that DLQI is highly specific for assessing decrements in QoL in patients with atopic dermatitis compared with the general population [51]. Specifically, patients with atopic eczema had a mean score of 4.2 compared to 0.3 in a normal population [50], [51]. DLQI has high repeatability, internal consistency, and sensitivity to change [51].

#### Infants' Dermatitis Quality of Life Index (IDQOL)

IDQOL was the fourth most common scale found in our review, used in 8 out of 382 RCTs (2.1%) examined. It was developed in 2001 by Drs. Lewis-Jones, Finlay, and Dykes to assess QoL in infants with AD [52]. IDQOL is completed by the parents of infants from birth to 4 years. The instrument consists of 10 questions regarding an infant or young child's difficulties with mood, sleep, bathing, dressing, play, mealtimes, other family activities, and treatment [52]. Each question is graded from 0–3 with a maximum total score of 30. A higher number correlates with a greater impairment of quality of life. An additional question exists that is scored separately on a scale of 0–4 that asks for the parents' overall assessment of eczema severity. In the original article the scale was validated with repeatability and sensitivity to change confirmed [52]. The scale was further validated with sensitivity to change confirmed and has been used in over 15 studies [43], [53].

## Discussion

Effective management of skin diseases begins with evaluation of both clinical disease severity and health-related QoL. In dermatology, the assessment of disease severity is frequently condition-specific and uses defined, observable parameters [54], [55], [56], [57]. QoL refers to the impact of a disease on a patient's overall function and wellbeing [58]. While disease severity is central for clinical evaluation and monitoring treatment response, QoL measures are as important for determining the effect of a disease or intervention on a patient's general welfare.

In the last 25 years, 20 disease-severity scales and 14 QoL instruments have been used in clinical trials involving patients with atopic dermatitis. Despite the emergence of multiple disease-severity and QoL instruments, few instruments have been validated. The four most commonly used disease-severity scales SCORAD, EASI, IGA, and SASSAD were used in 242 out of the 382 RCTs reviewed (63%). SCORAD, EASI, and SASSAD have been extensively validated [7]. The four most commonly used QoL instruments were DLQI, CDLQI, IDQOL, and DFI. All four scales have demonstrated validity, reliability, and sensitivity to change [36], [47], [51], [52].

The use of four top disease-severity instruments in AD has increased from 1985 to 2010 (Figure 2). For example, when we compared instrument usage patterns between the period from 2000–2004 with that from 2005–2010, we found that SCORAD usage increased by 106%; EASI usage increased by 165%; IGA usage increased by 169%, and SASSAD usage decreased by 20%. Over this ten-year period, IGA had the greatest rate of increase. The greater increased rate of IGA usage may be attributed to its ease of administration. Compared to IGA, EASI also experienced a higher rate of adoption in clinical trials since 1985. The usage patterns of EASI and IGA appear to parallel with each other, which suggests researchers' preference for both scales as objective measures of AD.

The increased usage of disease severity scales appeared to coincide with the publication of validation studies. SCORAD had its peak usage from 2005 to 2010, which corresponded closely to the publication of its validation studies from 2004 to 2006. EASI also had its peak usage from 2005 to 2010, which coincided with the publication of its validation studies in 2004 and 2005.

Among the four most commonly used QoL instruments, all were used more commonly as the years progressed. One possible explanation for this trend is that QoL measures have become as important as disease-severity instruments for patient evaluation and management. Of note, the four most common quality of life scales were developed by the same group of physicians and are similar in format and design. This may limit the diversity of the scale and the variety of characteristics that are used when assessing QoL in patients with atopic dermatitis. Additionally, our search of the literature was limited to randomized controlled trials. Other disease-specific quality of life instruments may exist that have not been used in randomized control trials.

Another limitation of our review is that, out of 382 RCTs examined, we identified 67 RCTs that used QoL measurements (18%), which makes identifying trends for individual QoL instruments difficult.

In this review, we identified trends for disease-severity and QoL outcomes measurements in atopic dermatitis from 1985–2010. We also summarized dimensions of the most commonly used scales and cited evidence for their validation. Although the consistent use of validated measures assessing disease severity and QoL in AD was not observed 20 years ago, this study found a promising trend of increased usage of validated instruments in clinical trials that measure AD disease severity and QoL in the past decade. Outcomes researchers in dermatology are encouraged to select validated outcomes measures that provide accurate measurement of disease dimensions and allow for comparison among studies.

This is the first systematic analysis of trends in the usage of outcomes measures in dermatological research. We anticipate that similar studies will be forthcoming in other disease areas within dermatology that assess the use of outcomes instruments. This type of study depicts the progression of a field's research quality and validity, and it encourages future outcomes researchers to devise instruments that will be streamlined, valid, and reliable.

## References

[1] Holm EA, Wulf HC, Stegmann H, Jemec GB (2006). Life quality assessment among patients with atopic eczema.. Br J Dermatol.

[2] Chamlin SL, Frieden IJ, Williams ML, Chren MM (2004). Effects of atopic dermatitis on young American children and their families.. Pediatrics.

[3] Hanifin JM, Rajka G (1980). Diagnostic features of atopic dermatitis.. Acta derm venereol (Stockh).

[4] Dohil MA, Eichenfield LF (2005). A treatment approach for atopic dermatitis.. Pediatr Ann.

[5] Finlay AY (1998). Quality of life assessments in dermatology.. Semin Cutan Med Surg.

[6] McKenna SP, Doward LC (2008). Quality of life of children with atopic dermatitis and their families.. Curr Opin Allergy Clin Immunol.

[7] Schmitt J, Langan S, Williams HC (2007). What are the best outcome measurements for atopic eczema? A systematic review.. J Allergy Clin Immunol.

[8] Kunz B, Oranje AP, Labreze L, Stalder JF, Ring J (1997). Clinical validation and guidelines for the SCORAD index: consensus report of the European Task Force on Atopic Dermatitis.. Dermatology.

[9] Charman C, Williams H (2000). Outcome measures of disease severity in atopic eczema.. Arch Dermatol.

[10] (1993). Severity scoring of atopic dermatitis: the SCORAD index. Consensus Report of the European Task Force on Atopic Dermatitis.. Dermatology.

[11] Angelova-Fischer I, Bauer A, Hipler UC, Petrov I, Kazandjieva J (2005). The objective severity assessment of atopic dermatitis (OSAAD) score: validity, reliability and sensitivity in adult patients with atopic dermatitis.. Br J Dermatol.

[12] Ben-Gashir MA, Seed PT, Hay RJ (2004). Quality of life and disease severity are correlated in children with atopic dermatitis.. Br J Dermatol.

[13] Breuer K, Braeutigam M, Kapp A, Werfel T (2004). Influence of pimecrolimus cream 1% on different morphological signs of eczema in infants with atopic dermatitis.. Dermatology.

[14] Charman CR, Venn AJ, Williams H (2005). Measuring atopic eczema severity visually: which variables are most important to patients?. Arch Dermatol.

[15] Hon KL, Kam WY, Lam MC, Leung TF, Ng PC (2006). CDLQI, SCORAD and NESS: are they correlated?. Qual Life Res.

[16] Hon KL, Leung TF, Wong Y, Fok TF (2006). Lesson from performing SCORADs in children with atopic dermatitis: subjective symptoms do not correlate well with disease extent or intensity.. Int J Dermatol.

[17] Hon KL, Ma KC, Wong E, Leung TF, Wong Y (2003). Validation of a self-administered questionnaire in Chinese in the assessment of eczema severity.. Pediatr Dermatol.

[18] Oranje AP, Stalder JF, Taieb A, Tasset C, de Longueville M (1997). Scoring of atopic dermatitis by SCORAD using a training atlas by investigators from different disciplines. ETAC Study Group. Early Treatment of the Atopic Child.. Pediatr Allergy Immunol.

[19] Pucci N, Novembre E, Cammarata MG, Bernardini R, Monaco MG (2005). Scoring atopic dermatitis in infants and young children: distinctive features of the SCORAD index.. Allergy.

[20] Schafer T, Dockery D, Kramer U, Behrendt H, Ring J (1997). Experiences with the severity scoring of atopic dermatitis in a population of German pre-school children.. Br J Dermatol.

[21] Staab D, Kaufmann R, Brautigam M, Wahn U (2005). Treatment of infants with atopic eczema with pimecrolimus cream 1% improves parents' quality of life: a multicenter, randomized trial.. Pediatr Allergy Immunol.

[22] Wolkerstorfer A, de Waard van der Spek FB, Glazenburg EJ, Mulder PG, Oranje AP (1999). Scoring the severity of atopic dermatitis: three item severity score as a rough system for daily practice and as a pre-screening tool for studies.. Acta Derm Venereol.

[23] Sprikkelman AB, Tupker RA, Burgerhof H, Schouten JP, Brand PL (1997). Severity scoring of atopic dermatitis: a comparison of three scoring systems.. Allergy.

[24] Fredriksson T, Pettersson U (1978). Severe psoriasis–oral therapy with a new retinoid.. Dermatologica.

[25] Hanifin JM, Thurston M, Omoto M, Cherill R, Tofte SJ (2001). The eczema area and severity index (EASI): assessment of reliability in atopic dermatitis. EASI Evaluator Group.. Exp Dermatol.

[26] Barbier N, Paul C, Luger T, Allen R, De Prost Y (2004). Validation of the Eczema Area and Severity Index for atopic dermatitis in a cohort of 1550 patients from the pimecrolimus cream 1% randomized controlled clinical trials programme.. Br J Dermatol.

[27] Belloni G, Pinelli S, Veraldi S (2005). A randomised, double-blind, vehicle-controlled study to evaluate the efficacy and safety of MAS063D (Atopiclair) in the treatment of mild to moderate atopic dermatitis.. Eur J Dermatol.

[28] Siegfried E, Korman N, Molina C, Kianifard F, Abrams K (2006). Safety and efficacy of early intervention with pimecrolimus cream 1% combined with corticosteroids for major flares in infants and children with atopic dermatitis.. J Dermatolog Treat.

[29] Berth-Jones J (1996). Six area, six sign atopic dermatitis (SASSAD) severity score: a simple system for monitoring disease activity in atopic dermatitis.. Br J Dermatol.

[30] Berth-Jones J, Finlay A, Zaki I, Tan B, Goodyear H (1996). Cyclosporine in severe childhood atopic dermatitis: A multicenter study* 1.. Journal of the American Academy of Dermatology.

[31] Granlund H, Erkko P, Sinisalo M, Reitamo S (1995). Cyclosporin in atopic dermatitis: time to relapse and effect of intermittent therapy.. Br J Dermatol.

[32] Berth-Jones J, Graham-Brown RA, Marks R, Camp RD, English JS (1997). Long-term efficacy and safety of cyclosporin in severe adult atopic dermatitis.. Br J Dermatol.

[33] Jenner N, Campbell J, Marks R (2004). Morbidity and cost of atopic eczema in Australia.. Australas J Dermatol.

[34] Sowden JM, Berth-Jones J, Ross JS, Motley RJ, Marks R (1991). Double-blind, controlled, crossover study of cyclosporin in adults with severe refractory atopic dermatitis.. Lancet.

[35] Charman CR, Venn AJ, Williams HC (2002). Reliability testing of the Six Area, Six Sign Atopic Dermatitis severity score.. Br J Dermatol.

[36] Lewis-Jones MS, Finlay AY (1995). The Children's Dermatology Life Quality Index (CDLQI): initial validation and practical use.. Br J Dermatol.

[37] Emerson RM, Lawson S, Williams HC (1998). Do specialist eczema clinics benefit children with atopic dermatitis?. British Journal of Dermatology.

[38] Harper JI, Ahmed I, Barclay G, Lacour M, Hoeger P (2000). Cyclosporin for severe childhood atopic dermatitis: short course versus continuous therapy.. Br J Dermatol.

[39] Aziah MS, Rosnah T, Mardziah A, Norzila MZ (2002). Childhood atopic dermatitis: a measurement of quality of life and family impact.. Med J Malaysia.

[40] Chuh AA (2003). Validation of a Cantonese version of the Children's Dermatology Life Quality Index.. Pediatr Dermatol.

[41] Clayton TH, Clark SM, Britton J, Pavlov S, Radev S (2007). A comparative study of the Children's Dermatology Life Quality Index (CDLQI) in paediatric dermatology clinics in the UK and Bulgaria.. J Eur Acad Dermatol Venereol.

[42] Holme SA, Man I, Sharpe JL, Dykes PJ, Lewis-Jones MS (2003). The Children's Dermatology Life Quality Index: validation of the cartoon version.. Br J Dermatol.

[43] Beattie PE, Lewis-Jones MS (2006). An audit of the impact of a consultation with a paediatric dermatology team on quality of life in infants with atopic eczema and their families: further validation of the Infants' Dermatitis Quality of Life Index and Dermatitis Family Impact score.. Br J Dermatol.

[44] Lewis-Jones M, Finlay A, Medicine WCo (2006). Quality of life research..

[45] Alvarenga TM, Caldeira AP (2009). Quality of life in pediatric patients with atopic dermatitis.. J Pediatr (Rio J).

[46] Grimalt R, Mengeaud V, Cambazard F (2007). The steroid-sparing effect of an emollient therapy in infants with atopic dermatitis: a randomized controlled study.. Dermatology.

[47] Lewis-Jones MS, Finlay AY, Dykes PJ (2001). The Infants' Dermatitis Quality of Life Index.. Br J Dermatol.

[48] Weber MB, Fontes Neto Pde T, Prati C, Soirefman M, Mazzotti NG (2008). Improvement of pruritus and quality of life of children with atopic dermatitis and their families after joining support groups.. J Eur Acad Dermatol Venereol.

[49] Finlay AY, Khan GK (1994). Dermatology Life Quality Index (DLQI)–a simple practical measure for routine clinical use.. Clin Exp Dermatol.

[50] Badia X, Mascaro JM, Lozano R (1999). Measuring health-related quality of life in patients with mild to moderate eczema and psoriasis: clinical validity, reliability and sensitivity to change of the DLQI. The Cavide Research Group.. Br J Dermatol.

[51] Lewis V, Finlay AY (2004). 10 years experience of the Dermatology Life Quality Index (DLQI).. J Investig Dermatol Symp Proc.

[52] Lewis-Jones MS, Finlay AY, Dykes PJ (1999). Measurement of the impact of atopic dermatitis on infant's and their families lives.. British Journal of Dermatology.

[53] Department of Dermatology WCoM, Cardiff University The Infant's Dermatitis Quality of Life Index (IDQOL)

[54] Chren MM (2000). Giving “scale” new meaning in dermatology: measurement matters.. Arch Dermatol.

[55] Williams H (1997). Is a simple generic index of dermatologic disease severity an attainable goal?. Archives of Dermatology.

[56] Chen SC (2007). Dermatology quality of life instruments: sorting out the quagmire.. J Invest Dermatol.

[57] Barzilai DA, Weinstock MA, Mostow EN (2007). Practicing evidence-based dermatology: a short guide.. Skinmed.

[58] Finlay AY (1997). Quality of life measurement in dermatology: a practical guide.. Br J Dermatol.

