# Correction: Health Outcome Measures in Atopic Dermatitis: A Systematic Review of Trends in Disease Severity and Quality-of-Life Instruments 1985–2010

**DOI:** 10.1371/annotation/6d5e99c5-bd8f-4cef-b77a-fbb795633da0

**Published:** 2011-06-02

**Authors:** Balvinder Rehal, April Armstrong

The second author's name should be: April W. Armstrong. The corrected citation is: Rehal B, Armstrong AW (2011) Health Outcome Measures in Atopic Dermatitis: A Systematic Review of Trends in Disease Severity and Quality-of-Life Instruments 1985 - 2010. PLoS ONE 6(4): e17520. doi:10.1371/journal.pone.0017520. The corrected Author Contributions are: Conceived and designed the experiments: AWA BR. Analyzed the data: AWA BR. Contributed reagents/materials/analysis tools: AWA BR. Wrote the paper: AWA BR. 

